# Patient safety and quality improvement: a ‘CLER’ time to move beyond peripheral participation

**DOI:** 10.3402/meo.v21.31993

**Published:** 2016-07-22

**Authors:** Daniel J. Schumacher, John G. Frohna

**Affiliations:** 1 Department of Pediatrics, Cincinnati Children's Hospital Medical Center, Cincinnati, OH, USA; 2 Department of Pediatrics, University of Wisconsin School of Medicine and Public Health, Madison, WI, USA; 3 Department of Medicine, University of Wisconsin School of Medicine and Public Health, Madison, WI, USA

**Keywords:** experiential learning, patient safety, quality improvement, residency training, graduate medical education

## Abstract

In the United States, the Accreditation Council for Graduate Medical Education (ACGME) has instituted a new program, the Clinical Learning Environment Review (CLER), that places focus in six important areas of the resident and fellow working and learning environment. Two of these areas are patient safety and quality improvement (QI). In their early CLER reviews of institutions housing ACGME-accredited training programs, ACGME has found that despite significant progress in patient safety and QI to date much work remains, especially when it comes to meaningful engagement of medical trainees in this work. In this article, the authors argue that peripheral involvement of trainees in patient safety and QI work does not allow the experiential learning that is necessary for professional development and the ultimate ability to execute performance that meets the needs of patients in contemporary clinical practice. Rather, as leaders in patient safety and QI have advocated since early in this movement, embedded and immersed experiences are necessary for learning and success.

In the United States, the Accreditation Council for Graduate Medical Education (ACGME) has instituted a Clinical Learning Environment Review (CLER) program that provides much needed focus on historically neglected components of the resident and fellow working and learning environment. While some of the six areas for CLER are now a common focus of training programs accredited by ACGME, such as duty hours, fatigue management, and care transitions, others remain a challenge. In reports of lessons learned during initial CLER visits, patient safety and quality improvement (QI) were unmistakable examples of the latter (1, 2). With reviews of nearly 300 sponsoring institutions that house nearly 9,000 ACGME-accredited training programs now complete, the first comprehensive report of CLER findings released this year maintains this tenor (3, 4). Notable findings in the report include 1) while most residents and fellows report formal training or education in patient safety, most also have limited knowledge in this area; 2) most clinical learning environments have few residents and fellows with working knowledge of QI; 3) less than half of the residents who have experienced patient safety concerns have reported these concerns, often relying on others to do so; and 4) when they do file patient safety reports, residents and fellows receive follow-up on less than half of these and are most often not included in any investigation of the event (3).

While these findings signal that many institutions still struggle to make meaningful headway in these areas, they also raise concern that institutions often do not engage residents and fellows in patient safety and QI, even at the level of competence for the institution. Indeed, one of the four overarching themes in the 2016 CLER report is the finding that graduate medical education efforts are often independent of other areas of strategic planning and focus within organizations (4). Given that patient safety and QI not only intimately involve the residents and fellows who are often marginalized in them but are also core competencies of physicians in contemporary health care, they should not be considered to be aspects of being a doctor that residents and fellows can learn later as attendings.

## When peripheral participation is ‘legitimate’

The approach of having layers of involvement in the work of a profession based on embedment in that community is not wrong. Rather, it is consistent with Lave and Wenger's concept of legitimate peripheral participation (5), where those with the least experience first participate in simple, lower risk tasks as they learn the territory (while these tasks are ‘peripheral’, they are still of overall benefit and thus ‘legitimate’) and then assume more and more responsibility commensurate with abilities and understanding of the community of practice. Common examples of this include the roles and activities of medical learners earlier in their training, such as a medical student working with radiology scheduling to coordinate an MRI for a patient he or she is caring for on the inpatient service. This task is just one piece of care that is likely not central to the patient's care. However, if the test is needed, it is still important. Thus, while this work is ‘peripheral’, it is still ‘legitimate’. It also allows the student to begin to learn how the system functions while matching the task to his or her current abilities. While it is clear that peripheral participation can be legitimate and developmentally appropriate, it is not clear how much of a resident's or fellow's time should be spent in patient safety and QI activities near the periphery. For those early in training, a significant proportion may be appropriate. If a resident is still determining his or her own role in patient care, it is understandable that he or she may not understand how that role interacts with others to make the system more efficient and reliable. Similarly, if he or she does not consistently identify medical errors or areas for system improvement, he or she may not understand the importance of filing safety reports aimed at creating solutions to safety threats, let alone have meaningful involvement in QI efforts.

## When peripheral participation is not legitimate: optimizing experiential learning

While some degree of peripheral participation may be appropriate for those early in training, two additional suppositions also seem likely: 1) this is not true for those in the later stages of training and 2) residents and fellows are unlikely to develop optimally in areas they do not train in. The latter statement is especially true if we believe, as Dewey and others have advocated, that learning is inextricably linked to the environment in which it takes place, with the experiences and interactions of individuals and environments constructing learning (6, 7). Thus, embedding residents and fellows *fully* in the practice of physicians and not just trainees during their training years is central to achieving the goals necessary for them to practice in contemporary care systems, which focus on patient safety and QI, without supervision. Stated differently, it is imperative to move residents and fellows beyond peripheral participation to full engagement in the real work of physicians in the real health care environment.

How, then, do institutional and program leaders train residents and fellows to be fully engaged in patient safety and QI? The answer may be deceptively simple. What is required of physicians in these areas is known, so these activities should be required of residents and fellows as well. While the answer is simple, doing this may not be easy based on what has been learned during CLER visits thus far. In their early experiences with CLER, the ACGME uncovered that ‘the role of residents in organizational efforts to improve health care quality and patient safety was, at best, uncertain’ (8). Furthermore, they have found that institutions vary in the ‘extent to which they invest in continually educating, training, and integrating faculty members and program directors in the areas of health care quality, patient safety, and other systems-based initiatives’ (2). Perhaps most concerning, they found that institutional and program leaders lack basic skills in understanding and ensuring patient safety and QI (6).

With these barriers, one might ask whether residents and fellows can experience meaningful learning in patient safety and QI in many current clinical learning environments. Even Dewey, a staunch advocate that ‘all genuine education comes about through experience’ also noted that ‘not all experiences are genuinely or equally educative’ (6, p. 25). So, where does one start in addressing this gap? We believe that there may be no other starting point than diving into doing QI work in the authentic clinical learning environment and learning how to do it better and better through trial and error. As Batalden and Davidoff note, ‘learning how to do quality improvement and actually carrying out quality improvement are essentially one in the same’ (9). A baseline skill set is necessary, but it is important to move quickly from this to continuing to build that skill set while actually engaging in efforts to make improvements. This approach is akin to the implementation of ACGME milestones, where it has been suggested that the plane is being built in flight (10, 11). Despite this perhaps less-than-ideal approach, the early improvements in assessment practices, outcomes, and scholarship that the Milestone project has spearheaded have been exceptionally noteworthy (12–14).

What, then, does building the plane of learning and doing QI in clinical learning environments in flight look like? From a broader viewpoint, we believe focus should be shifted from didactic sessions aimed at learning QI principles, such as completing QI modules, to learning QI in the clinical environment, such as engaging residents as key drivers, informed and engaged participants, and even leaders, of departmental and institutional QI (15). This could fill the gap uncovered in the 2016 CLER report, where many residents and fellows reported formal training in these areas (such as completing QI modules) but did not possess a working, practical knowledge of patient safety, and QI in the clinical environment. At a micro level, when a residents and fellows incorrectly writes a prescription, the pharmacy call to clarify the order should be directed to that trainee when possible. When an error involving a resident or fellow is uncovered, discussions about what went wrong and how similar errors can be prevented in the future should involve the resident and not just the attending faculty member. When nurses sidestep the resident to ask questions of the attending as an efficient work-around to achieve the specific aims of the departmental QI project, the resident and attending should redirect the nurse and emphasize the importance of engaging the resident for the sake of the resident's learning as well as optimizing the function of the systems in which residents are key components. If a resident or fellow files a safety report, it is imperative to not only follow-up with him but also include him in the discussion and investigation of the concern and how to make improvements based on what is discussed and learned. Meetings about flow and efficiency in a resident's continuity clinic should include the resident. Ad hoc e-mail conversations about communication breakdowns between services should include the resident or fellow involved in the care of the relevant patient and not just attending physicians or service chiefs. The list of possibilities for resident and fellow engagement is likely quite long, providing much opportunity for future work.

## A ‘CLER’ time to move beyond peripheral participation to embedded experiential learning

The patient safety and QI movement is flourishing. Despite this, the CLER program has found that residents, fellows, programs, and even institutions are often peripheral participants in this movement. Unless all of these stakeholders dive into learning and practicing patient safety and QI, learning and patient care in the authentic clinical learning and working environment cannot be optimized. Taking a first step may not be as hard as it seems. When learning to swim, one requires only enough activation energy to move to the edge of the pool, close one's eyes, and jump. Once immersed, it is impossible not to become wet. Similarly, once immersed, it is impossible not to begin to learn the principles and practice of patient safety and QI.
